# Thermoacoustic Sensing of Temperature Variations in Water-Saturated Porous Media

**DOI:** 10.3390/s26154709

**Published:** 2026-07-24

**Authors:** Chang Wang, Raquel Martinez Lopez, Chang Liu, Jose Angel Martinez-Lorenzo

**Affiliations:** Department of Mechanical and Industrial Engineering, Northeastern University, Boston, MA 02115, USA; wang.chang1@northeastern.edu (C.W.); martinezlopez.r@northeastern.edu (R.M.L.); liu.chang4@northeastern.edu (C.L.)

**Keywords:** thermoacoustic sensing, electromagnetic wave, porous media, temperature monitoring, multiphysics simulation, rock physics model

## Abstract

Accurate monitoring of temperature variations in fluid flow through porous media is important for numerous geophysical, environmental, and chemical processes. Traditional temperature measurement techniques often suffer from limitations such as invasiveness, restricted spatial coverage, or limited capability for real-time subsurface sensing. Thermoacoustic (TA) methods provide a promising alternative by combining electromagnetic excitation with acoustic detection to enable non-contact subsurface monitoring. In this work, thermoacoustic measurements were conducted over a temperature range of 20–60 °C using a water-saturated sand porous medium, and a multiphysics simulation framework incorporating rock physics models, temperature-dependent material properties, acoustic dispersion, and attenuation calibration was developed to reproduce the experimental observations. A clear relationship between temperature and TA signal amplitude was observed, and strong agreement was achieved between the experimentally measured and simulated thermoacoustic responses after applying the proposed calibration procedures. The results demonstrate the feasibility of using thermoacoustic signals to monitor bulk temperature variations in porous media and establish a foundation for future development of spatially resolved thermoacoustic temperature imaging methods.

## 1. Introduction

In situ chemical oxidation (ISCO) is a powerful remediation technology aimed at removing environmental contaminants from the soil. It has been successfully used to treat a wide range of volatile and semi-volatile hazardous substances. ISCO relies on redox (reduction/oxidation) reactions to chemically transform hazardous compounds into nonhazardous or less toxic forms that are more stable, less mobile, or inert [1,2]. Each chemical reaction releases or absorbs energy, leading to changes in enthalpy and, consequently, variations in temperature [3]. Thus, prolonged temperature changes in a specific area can indicate that a chemical reaction is taking place.

This study is essential for advancing the detection of chemical reactions by developing a method to sense temperature changes remotely, without direct contact with the target area. Detecting a temperature change serves as confirmation that a chemical reaction, and hence the remediation process, is occurring. This validation is crucial to ensure that the entire target area is being effectively treated and will be free of hazardous compounds once the remediation process is complete. The ability to monitor and verify ongoing chemical reactions through temperature variations ensures the efficiency and success of the ISCO process.

Previous research on ISCO modeling and monitoring has leveraged remote sensing data, particularly satellite imagery [4]. Other studies have relied on physical soil sampling to assess soil contamination [5,6]. However, there remains a gap in studies focusing on contactless, in situ monitoring of contaminated soil and remediation progress. This study not only enhances the detection of chemical reactions but also ensures thorough remediation of the target area, ultimately contributing to a cleaner and safer environment. This research represents a significant advancement in the field of ISCO.

Traditional contact-based methods, such as Electrical Resistivity Tomography (ERT) [7], the Self-Potential (SP) method [8], and Distributed Temperature Sensing (DTS) [9], cannot operate at standard distances. Non-contact methods like X-ray and infrared imaging, although widely used in thermal detection and processing, are limited in scope. X-ray imaging lacks real-time capabilities [10,11], while infrared imaging technologies, such as thermal cameras [12,13], are mainly used for surface inspections.

The main motivation behind this research is to enable non-invasive monitoring of temperature variations in porous media, which is important for understanding physical and chemical processes associated with subsurface remediation. During ISCO treatment, exothermic chemical reactions generate heat, making temperature a useful indicator of reaction activity and remediation progress. Continuous monitoring of temperature changes can therefore provide valuable information regarding treatment effectiveness and subsurface process evolution. However, the limitations of existing monitoring techniques highlight the need for alternative approaches capable of sensing temperature-dependent changes within porous media.

Thermoacoustic (TA) methods offer promising capabilities for subsurface information acquisition, with real-time TA imaging possible through the use of compressive sensing techniques and multi-element transducers [14].

Unlike conventional temperature sensors that provide measurements only at discrete locations, thermoacoustic methods are sensitive to temperature-dependent material properties, including thermal expansion, density, dielectric properties, and acoustic propagation characteristics. Consequently, thermoacoustic signals can potentially be used to monitor temperature-dependent behavior in porous media and provide additional insight into subsurface thermal processes.

In this work, a combined experimental and numerical investigation is performed to evaluate the feasibility of thermoacoustic temperature monitoring in water-saturated porous media. A multiphysics model is developed to simulate electromagnetic excitation, thermoacoustic generation, and acoustic wave propagation. The model incorporates temperature-dependent material properties derived from rock physics formulations, experimentally measured thermal expansion coefficients based on Kell’s thermophysical data, and temperature-dependent acoustic dispersion and attenuation calibrations. Controlled experiments are conducted between 20 °C and 60 °C using a water-saturated sand target to validate the numerical model.

The connection between thermoacoustic temperature monitoring and ISCO applications is particularly important. During ISCO treatment, reaction-induced temperature changes provide valuable information regarding reaction progress and treatment effectiveness. Although the present study is conducted under laboratory conditions, the proposed methodology establishes a framework for investigating temperature-dependent thermoacoustic responses in porous media. Future field-scale implementation will require consideration of electromagnetic penetration depth, geological heterogeneity, and site-specific material properties. Nevertheless, the ability to remotely monitor temperature-dependent thermoacoustic signals may provide a complementary approach to existing ISCO monitoring techniques such as thermocouples, distributed temperature sensing systems, and electrical resistivity methods.

The results demonstrate that the proposed framework successfully captures the temperature-dependent thermoacoustic response of water-saturated porous media. After incorporating experimentally measured acoustic dispersion, attenuation, and temperature-dependent material properties, the simulated thermoacoustic amplitudes show good agreement with the experimental measurements. The proposed approach therefore establishes the feasibility of thermoacoustic sensing as a tool for monitoring temperature variations in porous media and provides a foundation for future investigations of thermoacoustic monitoring in ISCO and other subsurface remediation applications.

The present study focuses on bulk temperature monitoring under controlled laboratory conditions. Therefore, the objective of this work is to verify the feasibility of monitoring temperature-dependent thermoacoustic responses in porous media rather than reconstructing spatial temperature distributions. Future work will investigate heterogeneous porous media, larger-scale targets, and spatially varying temperature distributions to further evaluate the applicability of thermoacoustic methods for field-scale subsurface monitoring.

## 2. Methodology

### 2.1. Main Theory

#### 2.1.1. TA Governing Equations

**Time domain equations**

Microwave-induced TA pressure waves are generated due to the thermal expansion of an object when it is illuminated by a short and intense microwave pulse. Considering a fixed microwave frequency *f*, the governing equations of TA wave generation in the time domain can be written as:

(1) $$\nabla \times \left(\nabla \times \bar{E}\left(r,w_{e}\right)\right)-k_{0}^{2}\left(w_{e}\right)\left(\epsilon_{r}\left(r,w_{e}\right)-\frac{j\sigma (r,w_{e})}{w_{e}\epsilon_{0}}\right)\bar{E}\left(r,w_{e}\right)=0$$

(2) $$\rho \left(r\right)\frac{\partial^{2}u\left(r,t\right)}{\partial t^{2}}=\nabla \cdot \bar{\bar{S}}\left(r,t\right)$$

(3) $$\rho \left(r\right)C_{p}\left(r\right)\frac{\partial T(r,t)}{\partial t}=\sigma \left(r\right){\left(∥\bar{E}\left(r\right)∥f\left(t\right)\right)}^{2}$$

(4) $$\nabla^{2}p\left(r,t\right)-\frac{1}{c_{l}^{2}\left(r\right)}\frac{\partial^{2}p\left(r,t\right)}{\partial t^{2}}=-\rho \left(r\right)\beta \left(r\right)\frac{\partial^{2}T\left(r,t\right)}{\partial t^{2}}$$

(5) $$\nabla^{2}p\left(r,t\right)-\frac{1}{c_{l}^{2}\left(r\right)}\frac{\partial^{2}p\left(r,t\right)}{\partial t^{2}}=-\frac{\beta (r)}{C_{p}\left(r\right)}\sigma \left(r\right){∥\bar{E}\left(r\right)∥}^{2}\frac{\partial [f^{2}\left(t\right)]}{\partial t}$$

where $w_{e}$ is the angular frequency of the electromagnetic wave, $\bar{E}\left(r,w_{e}\right)$ is the average electric field in the frequency domain, $k_{0}\left(w_{e}\right)$ is the free-space wavenumber, $\epsilon_{r}\left(r,w_{e}\right)$ is the relative permittivity, $\sigma (r,w_{e})$ is the electrical conductivity, $\epsilon_{0}$ is the vacuum permittivity, ρ(r) is the density, u(r,t) is the displacement field, $\bar{\bar{S}}\left(r,t\right)$ is the stress tensor, $C_{p}\left(r\right)$ is the specific heat capacity at constant pressure, T(r,t) is the temperature variation, *t* is time, f(t) is the microwave pulse function, p(r,t) is the TA pressure field, $c_{l}\left(r\right)$ is the longitudinal wave speed, and β(r) is the thermal expansion coefficient.

**Frequency domain equations**

To account for dispersion in porous media, a frequency-domain formulation is also used. The frequency-domain pressure field is written as:

(6) $$\nabla^{2}p\left(r,w_{a}\right)+\frac{w_{a}^{2}}{c_{l}^{2}\left(r\right)}p\left(r,w_{a}\right)=-jw_{a}\frac{\beta (r)}{C_{p}\left(r\right)}Q_{m}\left(r,w_{a}\right)\;$$

where $w_{a}$ is the angular frequency of the acoustic wave, $p(r,w_{a})$ is the TA pressure field in the frequency domain, and $Q_{m}\left(r,w_{a}\right)$ is the heat source term, defined as:

(7) $$Q_{m}\left(r,w_{a}\right)=\sigma \left(r\right){∥\bar{E}\left(r\right)∥}^{2}\left(f\left(w_{a}\right)*f\left(w_{a}\right)\right)\;$$

where $f(w_{a})$ is the frequency-domain response of the excitation signal and ∗ represents convolution.

According to Equation (7), the right-hand side of Equation (6) can be written as:

(8) $$S\left(r,w_{a}\right)=-jw_{a}\frac{\beta (r)}{C_{p}\left(r\right)}\sigma \left(r\right){∥\bar{E}\left(r\right)∥}^{2}\left(f\left(w_{a}\right)*f\left(w_{a}\right)\right)\;$$

This source term is composed of two parts: a frequency-dependent modulation term $-jw_{a}\left(f\left(w_{a}\right)*f\left(w_{a}\right)\right)$ and a material-dependent spatial distribution given by $\frac{\beta (r)}{C_{p}\left(r\right)}\sigma \left(r\right){∥\bar{E}\left(r\right)∥}^{2}$. The second term depends on the effective material properties of the porous medium and can be approximated using rock physics models.

Equation (6) is an inhomogeneous Helmholtz equation, and its solution can be written as:

(9) $$p\left(r,w_{a}\right)=-jw_{a}\left(f\left(w_{a}\right)*f\left(w_{a}\right)\right)\int_{\Omega }G\left(r,r^{\prime },w_{a}\right)\frac{\beta \left(r^{\prime }\right)\sigma \left(r^{\prime }\right){∥E(r^{\prime })∥}^{2}}{C_{p}\left(r^{\prime }\right)}\;dr^{\prime }\;$$

where $r^{\prime }$ is the source location, Ω is the imaging domain containing all TA sources, and $G(r,r^{\prime },w_{a})$ is the Green’s function computed by:

(10) $$\nabla^{2}G\left(r,r^{\prime },w_{a}\right)+k^{2}\left(r,w_{a}\right)G\left(r,r^{\prime },w_{a}\right)=-\delta \left(r-r^{\prime }\right)\;$$

Combining Equations (8) and (9), and discretizing the imaging domain Ω into $N_{p}$ voxels, the pressure as a function of frequency can be approximated as:

(11) $$p\left(r,w_{a}\right)=-jw_{a}\left(f\left(w_{a}\right)*f\left(w_{a}\right)\right)\sum_{i=1}^{N_{p}}G\left(r,r_{i}^{\prime },w_{a}\right)S\left(r_{i}^{\prime }\right)\;$$

The source term in Equation (8) is defined in the simulation as a monopole source $Q_{m}$, expressed in units of $s^{-2}$. The electromagnetic power dissipation density is given by:

(12) $$\sigma \left(r\right){∥\bar{E}\left(r\right)∥}^{2}=Q_{\mathrm{loss}}\;$$

where $Q_{\mathrm{loss}}$ is the total power dissipation density with units of $W/m^{3}$.

The pulse signal in the time domain is defined as:

(13) $$pulse\left(t\right)=\left\{\begin{array}{cc} 1, & t_{1}\leq t\leq t_{2},\\ 0, & \mathrm{otherwise}.\; \end{array}\right.$$

where $t_{1}$ is the initial transmission time of the pulse and $t_{2}$ is the final transmission time of the pulse. In this study, $t_{1}=3$ µs and $t_{2}=4\;$µs.

After Fourier transformation, the frequency-domain expression becomes:

(14) $$f\left(w_{a}\right)*f\left(w_{a}\right)=\frac{1}{-jw_{a}}\left(e^{-jw_{a}t_{2}}-e^{-jw_{a}t_{1}}\right)\;$$

Thus, the monopole source $Q_{m}$ is expressed as:

(15) $$Q_{m}\left(r,w_{a}\right)=-2\pi \frac{\beta (r)}{C_{p}\left(r\right)\rho \left(r\right)}Q_{\mathrm{loss}}\left(e^{-jw_{a}t_{2}}-e^{-jw_{a}t_{1}}\right)\;$$

**Near field and far field calculation**

The far field and near field of an antenna are two distinct regions characterized by the electromagnetic fields surrounding the antenna. The far field, also known as the Fraunhofer region, is sufficiently distant from the antenna such that the angular distribution of radiated energy does not change significantly with distance. For accurate measurements, it is important to place the target in the far field because the wave field has stabilized into a predictable radiation pattern.

The near field, or Fresnel region, is close to the antenna and is characterized by varying radiation patterns and field strengths. In this region, electromagnetic fields include reactive components and exhibit significant amplitude and phase variations, which makes quantitative measurements more challenging.

The transition distance from the near field to the far field can be estimated as:

(16) $$R=\frac{2D^{2}}{\lambda }\;$$

where *R* is the far-field distance, *D* is the largest dimension of the antenna, and λ is the wavelength of radiation. From Equation (16) and the diameter of the circular transducer, the far-field distance can be calculated. Therefore, the target was positioned at a distance greater than *R* to obtain reliable experimental data.

To determine the pressure in the far field, the Helmholtz theorem is employed to transform the volume integral in Equation (9) into the surface integral representation expressed in Equations (17) and (18). This simplification allows the calculation to be confined to the pressure and pressure gradient on the surface of a domain enclosing the target. The pressure calculation is governed by:

(17) $$p\left(r^{\prime }\right)=\int_{S(r)}\left(G\left(r,r^{\prime }\right)\nabla p\left(r\right)-\nabla G\left(r,r^{\prime }\right)p\left(r\right)\right)\cdot n\;dS$$

(18) $$G\left(r,r^{\prime }\right)=\frac{e^{-jk_{a}\left|r-r^{\prime }\right|}}{4\pi |r-r^{\prime }|}$$

where n is the outward normal vector of the surface enclosing the target, r is the target location, $r^{\prime }$ is the receiver location, and $|r-r^{\prime }|$ is the distance between the target and the receiver.

#### 2.1.2. Rock Physics Model

The Rock Physics Models (RPM) provide analytical expressions for the effective constitutive properties of porous media.

The Bruggeman–Hanai–Sen (BHS) model and Krief’s model were selected to estimate the effective dielectric and elastic properties of the water-saturated porous medium, respectively. The BHS model has been extensively validated for heterogeneous geological materials and accounts for the interaction between fluids and the solid matrix through an effective-medium formulation [15]. Therefore, it provides a suitable representation of the dielectric behavior of fluid-saturated porous media. For the elastic properties, Krief’s model was adopted because it explicitly relates porosity to the bulk and shear moduli of porous materials and has been widely used in rock physics studies of granular and sedimentary media [16]. Compared with simple volumetric averaging approaches, the BHS and Krief models provide a more physically representative description of porous media while requiring substantially fewer material parameters than full poroelastic formulations, such as Biot theory [17]. Since thermoacoustic signal generation depends on the electromagnetic, thermal, and elastic properties of the target, porosity plays an important role in determining the effective material properties through these rock physics relationships.

The effective properties of the water-saturated porous medium are calculated using the following rock physics relationships. The mixed complex dielectric constant of water-saturated sand is described by the Bruggeman–Hanai–Sen model:

(19) $$\epsilon_{c}=\epsilon_{r}-j\frac{\sigma }{w_{e}}\;$$

where $\epsilon_{c}$ is the complex dielectric constant, $\epsilon_{r}$ is the relative permittivity, and σ is the electrical conductivity. The subscripts ${\left[-\right]}_{1}$ and ${\left[-\right]}_{2}$ denote the fluid and solid phases, respectively.

(20) $$\Phi =\frac{\epsilon_{c}-\epsilon_{c2}}{\epsilon_{c1}-\epsilon_{c2}{\left(\frac{\epsilon_{c1}}{\epsilon_{c}}\right)}^{1/3}}$$

where Φ is the porosity, $\epsilon_{c1}$ is the complex dielectric constant of the filling fluid, and $\epsilon_{c2}$ is the complex dielectric constant of the rock matrix.

The effective thermal expansion coefficient and specific heat capacity are calculated as:

(21) $$\beta =\frac{\left(1-\Phi \right)K_{2}\beta_{2}+\Phi K_{1}\beta_{1}}{\left(1-\Phi \right)K_{2}+\Phi K_{1}}\;$$

(22) $$C_{p}=\frac{\rho_{2}C_{p2}\left(1-\Phi \right)+\rho_{1}C_{p1}\Phi }{\rho_{2}}$$

where $K_{1}$ and $K_{2}$ are the bulk moduli of the fluid and rock phases, respectively, $\beta_{1}$ and $\beta_{2}$ are the thermal expansion coefficients of the fluid and rock phases, respectively, $C_{p1}$ and $C_{p2}$ are the specific heat capacities of the fluid and rock phases, respectively, and $\rho_{1}$ and $\rho_{2}$ are the densities of the fluid and rock phases.

As mentioned previously, the effective elastic properties are calculated using Krief’s model:

(23) $$\rho =\left(1-\Phi \right)\rho_{2}+\Phi \rho_{1}$$

(24) $$\beta_{B}=1-{\left(1-\Phi \right)}^{\frac{3}{1-\Phi }}$$

(25) $$K=K_{2}\left(1-\beta_{B}\right)+\beta_{B}^{2}M$$

(26) $$\mu =\mu_{2}\left(1-\beta_{B}\right)$$

(27) $$\frac{1}{M}=\frac{\beta_{B}-\Phi }{K_{2}}+\frac{\Phi }{K_{1}}$$

(28) $$C_{l}=\sqrt{\frac{K+\frac{4}{3}\mu }{\rho }}$$

where $\beta_{B}$ is the Biot coefficient, *M* is an intermediate elastic parameter, $\mu_{2}$ is the shear modulus of the rock matrix, μ is the effective shear modulus, *K* is the effective bulk modulus, and $C_{l}$ is the effective longitudinal wave velocity.

### 2.2. Dispersion Calibration in Porous Medium

**Figure 1:**
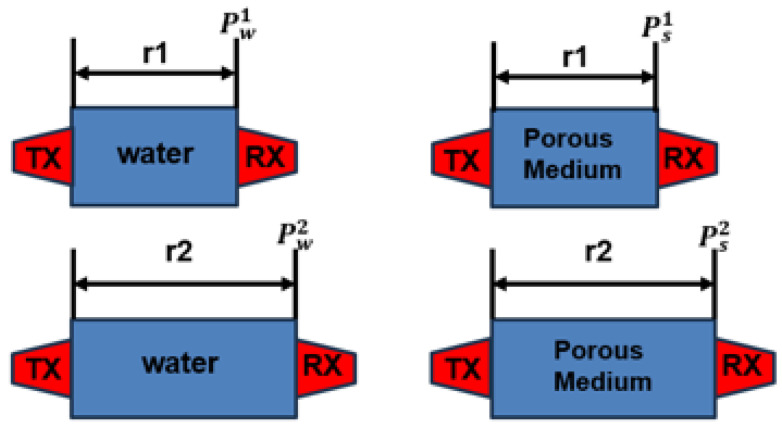
Dispersion Calibration Process.

According to previous studies on acoustic wave propagation, velocity dispersion in porous media must be considered. This effect varies with the materials such as different grain sizes, elastic properties, and packing procedures. The calibration procedures were introduced by Lee in 2009 [18]. In the simulation, the default setting of propagation velocity is constant, which does not accurately represent the experimental conditions. In order to match the propagation velocity in the simulation and experiments, a dispersion calibration experiment was conducted to compensate for the errors in simulation. The experiments include the water part and water-saturated sand part. The water measurements were conducted as a reference because the acoustic wave velocity in water is nearly frequency independent over the investigated frequency range. The measured acoustic pressure P(r,w) can be expressed by Equation (29) while the distance is large enough compared to the length of the wave.

(29) $$P\left(r,\omega \right)=\frac{A(\omega )}{4\pi \left|r-r^{\prime }\right|}e^{-j\frac{\omega }{c(\omega )}\left|r-r^{\prime }\right|}e^{-\alpha \left(\omega \right)\left|r-r^{\prime }\right|}\;$$

where A(w) is the point source strength, c(w) is the sound speed and α(w) is the attenuation factor. For this case, c(w) needs to be considered in the simulation. The dispersion calibration process is shown in Figure 1.

The acoustic wave is transmitted by transmitter and propagate through the medium. The acoustic wave after propagation is received by receiver. $P_{w}^{1}$ and $P_{w}^{2}$ denote the measured pressure of water with distance $r_{1}$ and $r_{2}$ while $P_{s}^{1}$ and $P_{s}^{2}$ is denoting the measured pressure of water saturated sand with distance $r_{1}$ and $r_{2}$. Thus, the measured pressures can be substituted into Equation (30).

(30) $$\begin{array}{cc} P_{w}^{1}\left(\omega \right) & =\frac{A(\omega )}{4\pi r_{1}}e^{-j\frac{\omega }{c_{w}\left(\omega \right)}r_{1}}e^{-\alpha_{w}\left(\omega \right)r_{1}}\\ P_{w}^{2}\left(\omega \right) & =\frac{A(\omega )}{4\pi r_{2}}e^{-j\frac{\omega }{c_{w}\left(\omega \right)}r_{2}}e^{-\alpha_{w}\left(\omega \right)r_{2}}\\ P_{s}^{1}\left(\omega \right) & =\frac{A(\omega )}{4\pi r_{1}}e^{-j\frac{\omega }{c_{s}\left(\omega \right)}r_{1}}e^{-\alpha_{s}\left(\omega \right)r_{1}}\\ P_{s}^{2}\left(\omega \right) & =\frac{A(\omega )}{4\pi r_{2}}e^{-j\frac{\omega }{c_{s}\left(\omega \right)}r_{2}}e^{-\alpha_{s}\left(\omega \right)r_{2}} \end{array}$$

where $c_{w}\left(w\right)$ is the phase speed for water, $c_{s}\left(w\right)$ is the phase speed for the porous medium, $\alpha_{w}\left(w\right)$ is the attenuation factor for water, and $\alpha_{s}\left(w\right)$ is the attenuation factor for the porous medium. Based on Equation (30), the phase speed and attenuation factor of sand can be represented as

(31) γ(ω)=tan−1ImPs2(ω)/Ps1(ω)RePs2(ω)/Ps1(ω),cs(ω)=ω(r2−r1)γ(ω)+nπ,αs(ω)=1r2−r1lnPw2(ω)Ps1(ω)Ps2(ω)Pw1(ω).

where nπ compensates for the phase difference due to wave propagation distance. In Equation (31), it is assumed that the attenuation factor of water is 0 and r2>r1.

**Figure 2:**
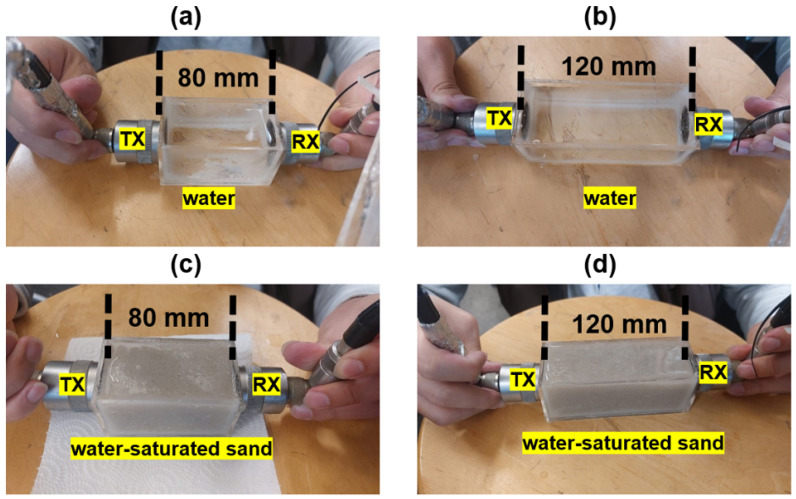
Dispersion calibration experiments: (**a**) Water in 80 mm length container; (**b**) Water in 120 mm length container; (**c**) water-saturated sand in 80 mm length container; (**d**) water-saturated sand in 120 mm length container.

The experiment procedure for the propagation medium calibration is shown in Figure 2. Two acrylic containers of different lengths (80 mm and 120 mm) are prepared, and these containers are filled with water-saturated sand or water during the experiment. The cross-section of the container is 50 mm × 50 mm. Two transducers are pressed against the container: one serving as the transmitting transducer (Tx) and the other one as the receiving transducer (Rx). The same acoustic waveform is transmitted for water and water-saturated sand in this experiment.

**Figure 3:**
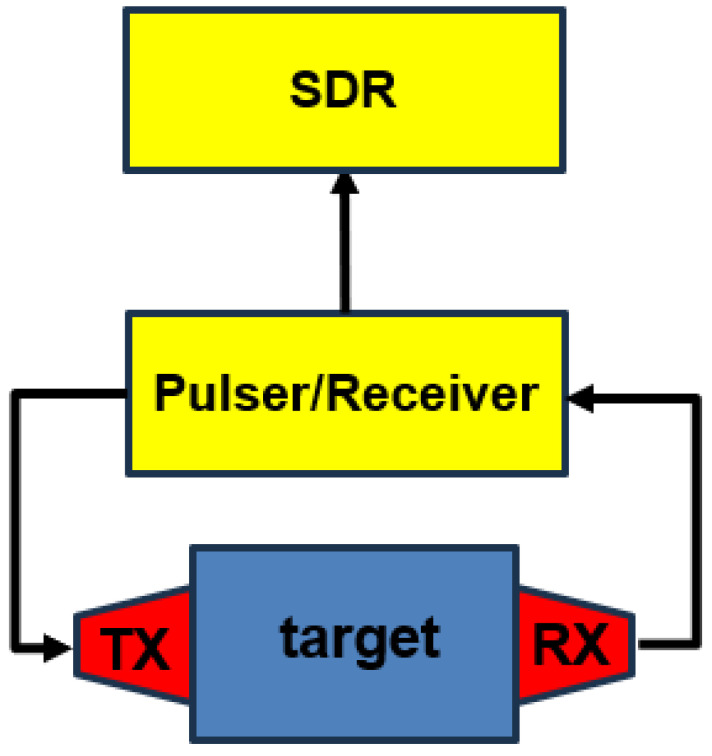
Cable connection for dispersion calibration experiments.

The cable connection pattern of different devices is presented in Figure 3.

**Figure 4:**
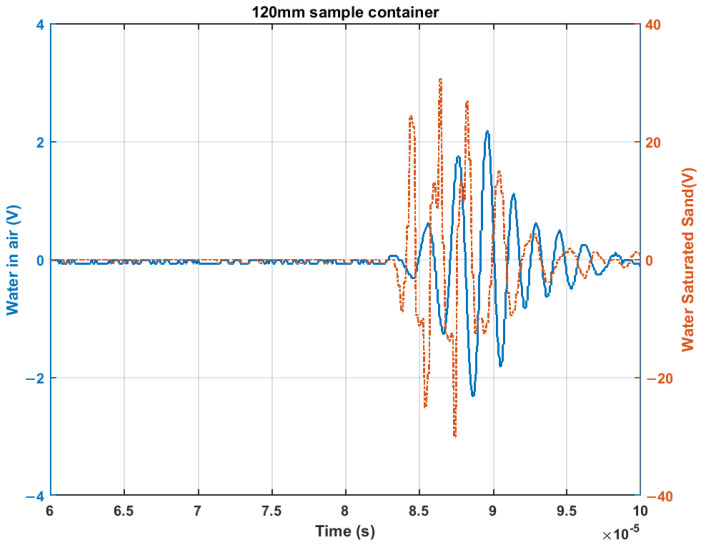
The comparison between the received acoustic measurements through water-saturated sand and water after propagating through a 120 mm sample.

**Figure 5:**
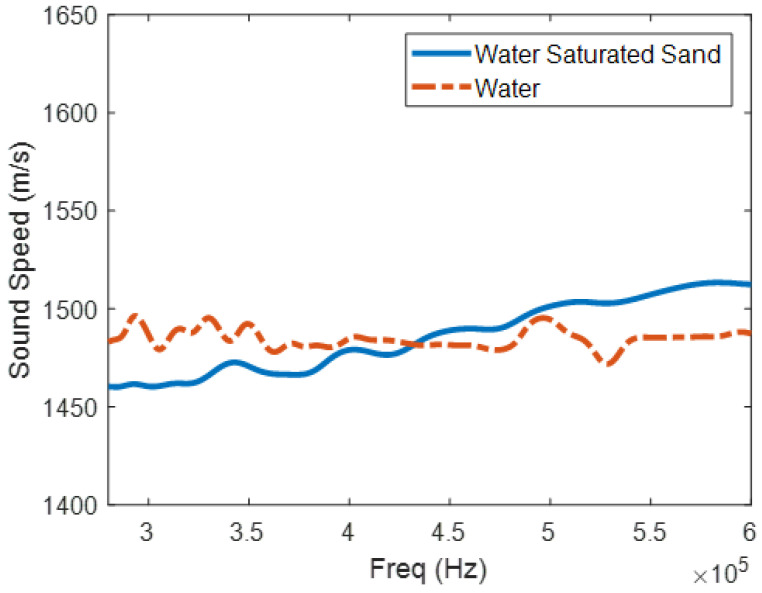
Phase velocity comparison between water and water-saturated sand.

Figure 4 presents the comparison between the received measurements after the acoustic wave propagating 120 mm through the water and water-saturated sand medium. It is shown that the acoustic wave arrives almost at the same time when traveling in the water and water-saturated sand, which indicates a similar phase velocity of water and water-saturated sand. Figure 5 shows the comparison between the derived phase velocity of water and water-saturated sand. It is observed that the phase velocity of water is almost a constant value of approximately 1480 m/s, which agrees well with the known acoustic properties of water. The phase velocity of water-saturated sand similarly starts from approximately 1460 m/s and slightly increases to 1520 m/s. In our U-shaped target, the width is only 6 mm, corresponding to a maximum propagation time deviation on the order of $10^{-9}$ s.

### 2.3. Temperature-Dependent Dispersion Calibration

Although the room-temperature measurements indicate that the influence of phase velocity dispersion is relatively small for the investigated target dimensions, temperature-dependent acoustic properties are required in order to incorporate dispersion effects into the thermoacoustic simulations. Therefore, additional dispersion calibration experiments were conducted at 20 °C, 30 °C, 40 °C, 50 °C, and 60 °C.

**Figure 6:**
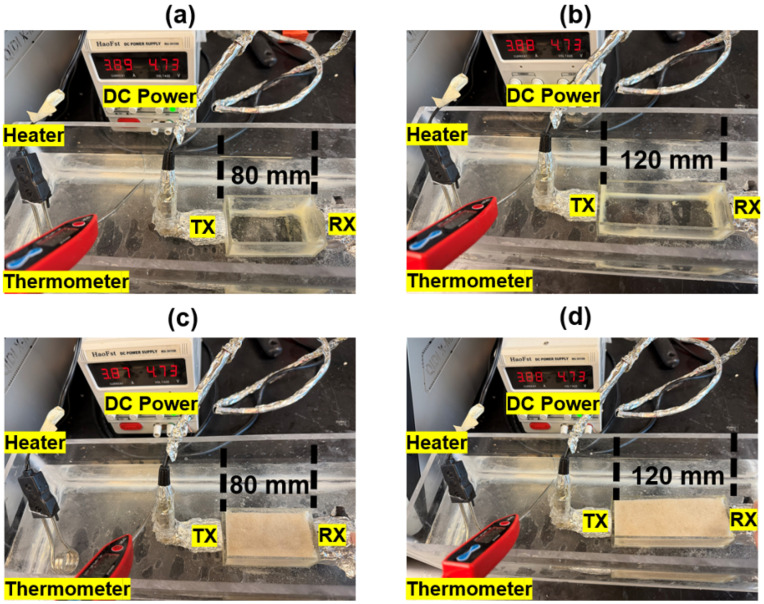
Experimental setup for temperature-dependent dispersion calibration: (**a**) water with an 80 mm propagation distance; (**b**) water with a 120 mm propagation distance; (**c**) water-saturated sand with an 80 mm propagation distance; and (**d**) water-saturated sand with a 120 mm propagation distance. The heater and DC power supply were used to control the medium temperature, while the thermometer was used to monitor the temperature during the measurements.

The experimental setup is shown in Figure 6. During the measurements, a heater powered by a DC power supply was used to gradually increase the temperature of the medium. A digital thermometer was employed to continuously monitor the medium temperature, and data acquisition was performed only after the temperature had stabilized at the desired value. The same measurement procedure described in the previous section was repeated for each temperature condition.

**Figure 7:**
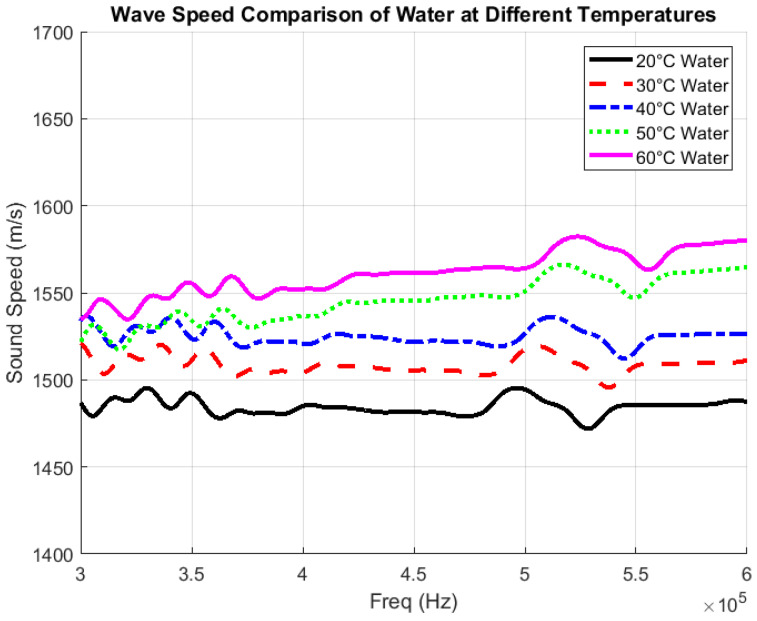
Frequency-dependent phase velocity of water at different temperatures.

**Figure 8:**
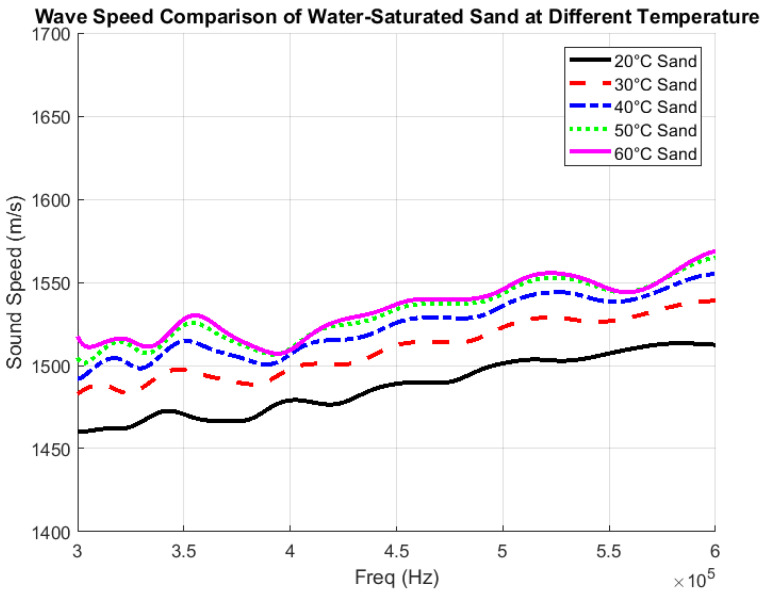
Frequency-dependent phase velocity of water-saturated sand at different temperatures.

The resulting frequency-dependent phase velocities of water and water-saturated sand are presented in Figure 7 and Figure 8, respectively. For both media, the phase velocity increases with increasing temperature over the investigated frequency range. In addition, moderate frequency-dependent dispersion can be observed at all temperatures.

**Figure 9:**
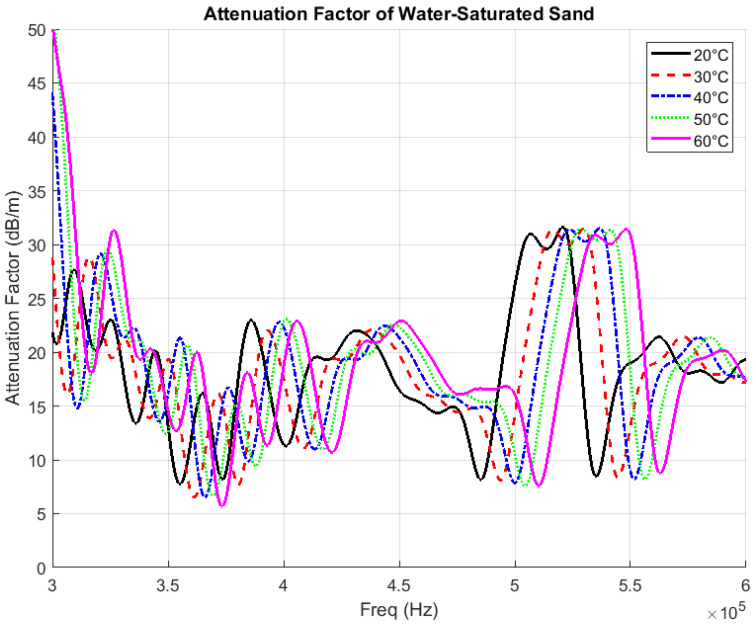
Frequency-dependent attenuation factor of water-saturated sand at different temperatures.

Figure 9 shows the attenuation factors derived from the water-saturated sand measurements at different temperatures. The attenuation exhibits both frequency-dependent and temperature-dependent variations over the investigated frequency range.

The measured temperature-dependent phase velocities were subsequently incorporated into the COMSOL Multiphysics 6.0 simulations through interpolation functions, while the measured attenuation factors were applied to the simulated frequency-domain acoustic responses during post-processing. This procedure allows the experimentally measured temperature-dependent acoustic behavior of the porous medium to be incorporated into the thermoacoustic model.

### 2.4. Simulation Model

**Figure 10:**
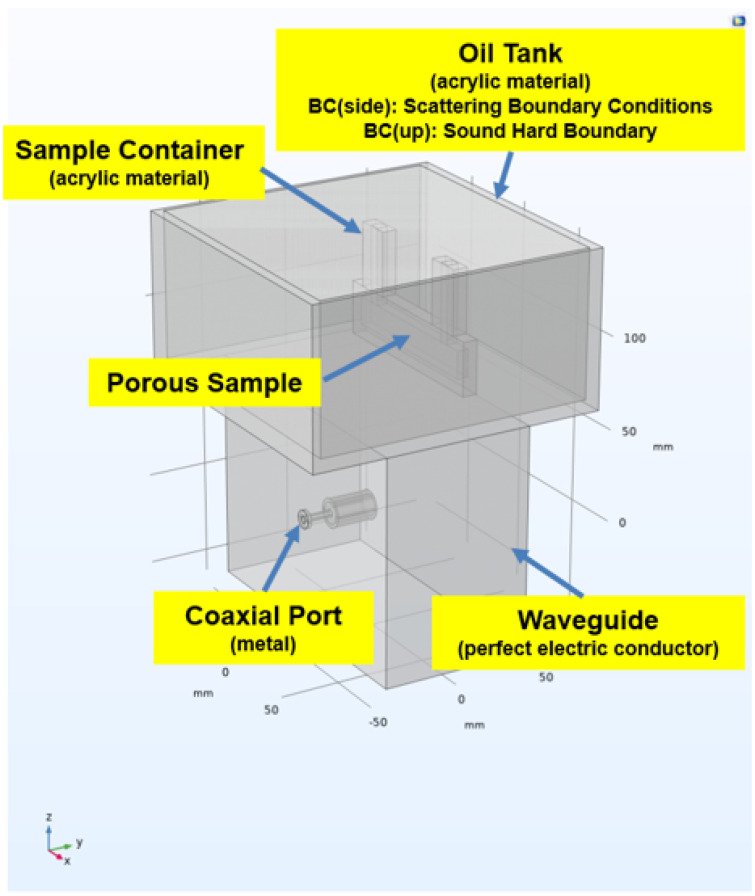
COMSOL Multiphysics 6.0 multiphysics simulation geometry used for thermoacoustic modeling.

To validate the results of the experiments, simulations were performed. These simulations replicate the same conditions and geometry as the actual experiments. A multiphysics thermoacoustic model was developed in COMSOL Multiphysics 6.0 using the same geometry and dimensions as the experimental setup, as shown in Figure 10. The model was used to compare the simulated and experimentally measured thermoacoustic responses. The lower section consists of a WR-650 waveguide (Pasternack Enterprises, Inc., Irvine, CA, USA) excited through a metallic coaxial cable, while the upper section contains the oil tank and the water-saturated sand target immersed in the coupling oil. The waveguide walls were modeled as perfect electric conductors (PEC), and the excitation port was driven with an input power of 2100 W, corresponding to the average RF amplifier output used during the experiments. The oil tank was filled with oil, while the container walls and supporting structures were modeled using acrylic material.

The electromagnetic field distribution was solved using the Electromagnetic Waves, Frequency Domain interface. The absorbed electromagnetic energy within the target region was converted into a thermoacoustic source according to Equation (15), thereby coupling the electromagnetic and acoustic physics. The generated acoustic pressure field was subsequently solved using the Pressure Acoustics, Frequency Domain interface over the frequency range from 0.28 MHz to 0.60 MHz, corresponding to the dominant frequency components observed in the experimental measurements.

The frequency-dependent phase velocity of the water-saturated sand at different temperatures was obtained from the dispersion calibration procedure described in Section 2.3. The calibrated phase velocity data were imported into COMSOL Multiphysics 6.0 through a Global Interpolation Function and assigned to the porous medium acoustic properties. As a result, the experimentally measured dispersion behavior was directly incorporated into the thermoacoustic forward model.

The frequency-dependent attenuation factor obtained from the dispersion calibration measurements was not incorporated directly into the COMSOL Multiphysics 6.0 acoustic solver. Instead, after the acoustic pressure spectrum was calculated in the frequency domain, the simulated spectrum was corrected in MATLAB (R2023b) using the experimentally measured attenuation factor. This approach preserves the measured attenuation characteristics while avoiding additional numerical damping within the finite-element model.

For the acoustic domain, the side walls of the oil container were assigned Scattering Boundary Conditions to reduce artificial reflections from the computational boundaries and better approximate an open acoustic environment. The upper surface of the oil tank was modeled as a Sound Hard Boundary due to the large acoustic impedance mismatch between the oil and the surrounding air. These boundary conditions provide a realistic representation of the experimental environment and enable direct comparison between the simulated and measured thermoacoustic responses.

The resulting multiphysics model captures the complete process of electromagnetic excitation, thermoacoustic source generation, frequency-dependent acoustic wave propagation, and attenuation correction, thereby providing a physically consistent framework for comparison with the experimental measurements.

Several material properties are required for the thermoacoustic simulations. Because the experiments were conducted at different temperatures, the temperature-dependent properties of both water and sand were first defined. The effective properties of the water-saturated sand were then calculated using the rock physics relationships described in Section 2.1.2.

The intrinsic properties of sand were assumed to remain constant over the temperature range investigated in this study because the thermal variation of quartz-based sand is negligible between 20 °C and 60 °C. The corresponding sand properties used in the simulations are summarized in Table 1. In contrast, several properties of water, including relative permittivity, density, electrical conductivity, thermal expansion coefficient, and acoustic wave velocity, vary significantly with temperature, as summarized in Table 2. Since the effective properties of the water-saturated porous medium depend on both the solid matrix and the pore fluid, a rock physics model was employed to calculate the temperature-dependent effective properties of the sample.

**Figure 11:**
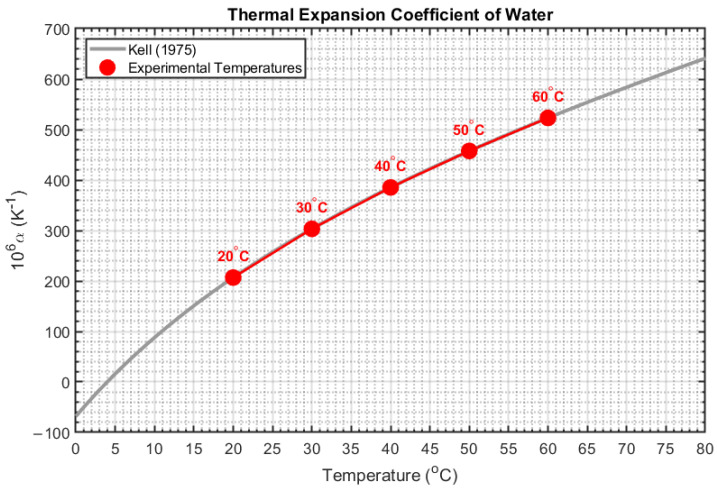
Temperature-dependent thermal expansion coefficient of water obtained from the experimental data reported by Kell [19]. The highlighted points correspond to the temperatures investigated in this study.

The thermal expansion coefficient plays an important role in thermoacoustic pressure generation and therefore must be accurately represented in the simulation model. In this study, the temperature-dependent thermal expansion coefficient of water was obtained from the experimental thermophysical data reported by Kell [19]. Figure 11 shows the variation of the thermal expansion coefficient over the temperature range considered in this work. The highlighted points correspond to the experimental temperatures investigated in this study. The corresponding thermal expansion coefficients at each temperature were incorporated into the water material properties and subsequently used in the rock physics model to calculate the effective thermoelastic properties of the water-saturated sand. This approach allows the thermoacoustic simulations to directly account for the influence of temperature on the thermal expansion behavior of the porous media using experimentally measured thermophysical data.

**Figure 12:**
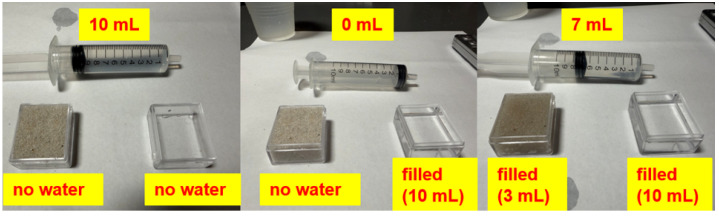
Experimental porosity estimation using the volumetric saturation method.

The porosity value used in the simulations was determined experimentally using a volumetric saturation method, as shown in Figure 12. Two identical containers were prepared: an empty container and a container filled with dry sand. The total container volume was first determined by filling the empty container with water, yielding a volume of approximately 10 mL. Water was then injected into the sand-filled container until the pore space was completely saturated, requiring approximately 3 mL of water. The porosity was therefore estimated as

(32) $$\phi =\frac{V_{\mathrm{pore}}}{V_{\mathrm{total}}}=\frac{3\;\mathrm{mL}}{10\;\mathrm{mL}}=0.30.$$

To evaluate the repeatability of the measurement, the porosity estimation was repeated ten times using independently prepared sand samples. The results are summarized in Table 3. The measured injected water volume ranged from 2.9 mL to 3.1 mL, corresponding to porosity values between 29% and 31%. The average porosity was approximately 30%, with an estimated uncertainty of ±1%. Therefore, a porosity value of 30% was adopted for all simulations. This value is also consistent with the typical porosity range reported for packed sand materials.

Porosity plays an important role in thermoacoustic signal generation because it directly influences the effective dielectric, thermal, and elastic properties of the porous medium through the rock physics model. Consequently, variations in porosity affect both microwave absorption and acoustic propagation characteristics. Previous studies have also reported that thermoacoustic signal amplitude increases with increasing water saturation in geological materials [20]. Since higher porosity generally allows a larger pore volume to be occupied by water, porous media with higher porosity and water content are expected to exhibit stronger thermoacoustic responses. Although porosity was fixed at 30% in the present study, these observations highlight the importance of incorporating porosity into the numerical model when predicting thermoacoustic behavior in porous media.

The elastic parameters calculated using the rock physics model are summarized in Table 4. The bulk modulus of water was calculated at each temperature from the measured density and longitudinal wave velocity, while the bulk and shear moduli of the sand skeleton were calculated from the measured longitudinal and shear wave velocities. Because the temperature dependence of quartz-based sand is relatively small over the investigated temperature range (20–60 °C), the elastic properties of the sand skeleton were assumed to remain constant. In contrast, the elastic properties of the fluid water vary significantly with temperature and were therefore updated for each temperature condition. These temperature-dependent fluid properties were incorporated into the rock physics model to calculate the effective elastic properties of the water-saturated sand. The resulting effective thermophysical, dielectric, and acoustic properties used in the thermoacoustic simulations are summarized in Table 5.

### 2.5. Experiment Validation

**Figure 13:**
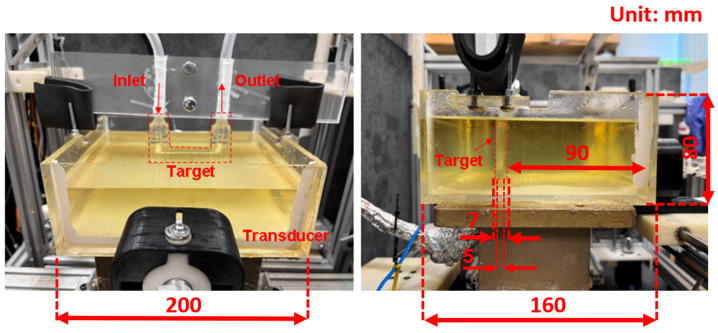
Experiment testbed.

**Figure 14:**
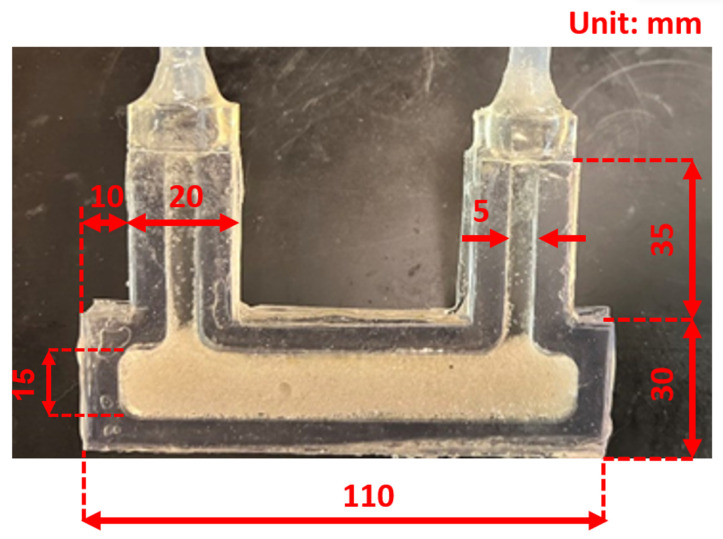
U-shaped target filled with porous sand.

A theoretical simulation based on solid mechanics and TA elastic wave governing equation was developed. Also, the multi-materials properties were calculated by the formulae indicated at Section 2.1.2 for the simulations with different temperatures. The selected microwave frequency for the experiment is 1.3 GHz, which is generated by the microwave source and amplified by a high-power microwave amplifier. The pulse repetition rate and pulse width are controlled by a function generator connected to the amplifier’s trigger input port. A WR-650 rectangular waveguide (Pasternack Enterprises, Inc., Irvine, CA, USA), filled with air, is connected to the amplifier to excite the 1.3 GHz microwave, with an oil container placed above it. The U-shaped sample is immersed in the oil container, 90 mm from the transducer. Conducted on the multi-physics simulation platform COMSOL Multiphysics 6.0, the simulation evaluates the TA performance of a water-saturated sand sample. In order to do the experiments, a U-shaped target filled with water and porous sand medium was built, containing the inlet and outlet for the fluid (Figure 13). The target is prepared with shape and dimensions shown in Figure 14. Furthermore, a fluid cycling system was established to accurately control the fluid temperature inside the target. In specific, the fluid is heated by an immersion heater and transported to the U-shaped target using a pump. Meanwhile, the U-shaped target was set to be immersed into the oil container, which was filled with oil to guarantee the far-field conditions of the thermoacoustic transducer.

**Figure 15:**
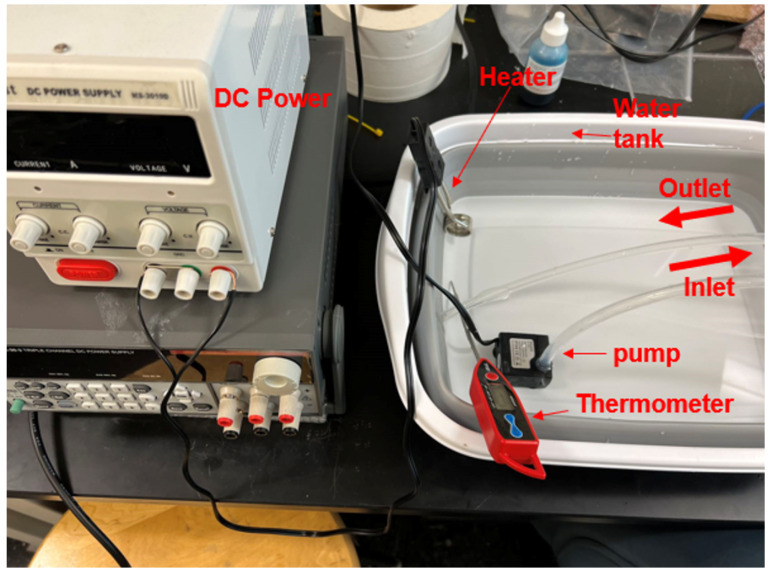
Temperature control system used for thermoacoustic experiments.

A dedicated fluid circulation and temperature control system was developed to ensure stable and repeatable temperature conditions during the experiments, as shown in Figure 15. The water inside the U-shaped target was heated using an immersion heater powered by a programmable DC power supply. A circulation pump continuously transported the heated water through the target to maintain a uniform temperature distribution. Real-time temperature measurements were monitored using digital temperature sensors and recorded through the data acquisition system.

**Figure 16:**
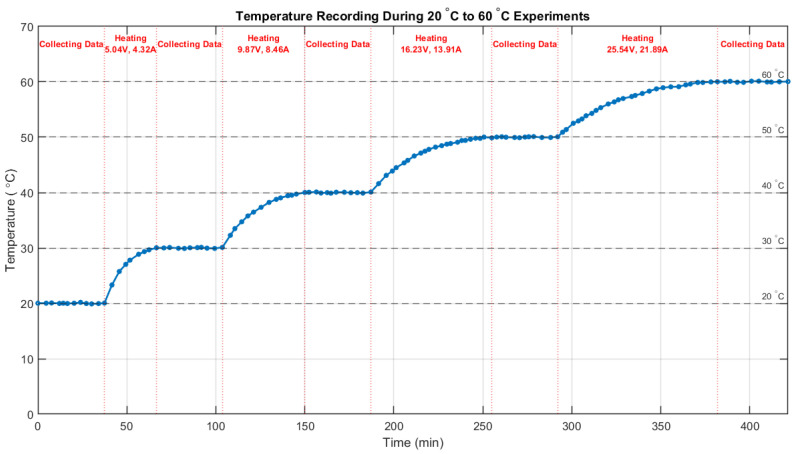
Temperature control performance during the experiments.

Figure 16 presents the measured temperature history during the experiments. For each temperature level, the heating voltage supplied by the DC power source was adjusted to achieve the desired temperature. After reaching the target temperature, the system was allowed to stabilize before data acquisition. The results demonstrate that each temperature stage remained stable for an extended period, providing sufficient time for thermoacoustic signal collection and minimizing uncertainty associated with temperature fluctuations.

**Figure 17:**
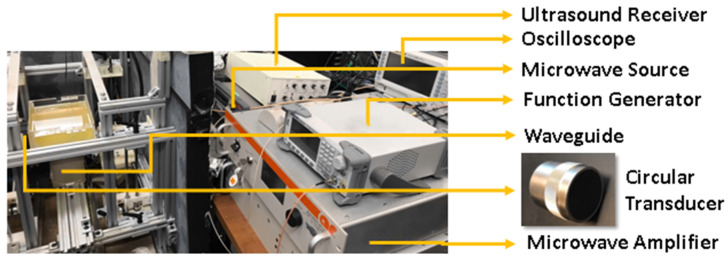
Experiment devices and elements.

**Figure 18:**
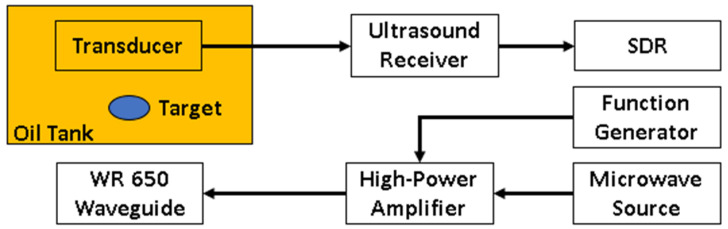
Diagram of the connections between devices.

The container measurements can be seen in Figure 13. Both the target and the container are made from laser-cut acrylic boards and sealed with waterproof silicone. For reliability, a single circular transducer Olympus A301 (Olympus NDT, Waltham, MA, USA) is used to collect the generated TA signal. The received TA measurement is filtered by a DPR300 ultrasonic pulser/receiver (JSR Ultrasonics, Pittsford, NY, USA). The sample is positioned in the oil tank, 90 mm away from the transducer, with the side and top views of the sample’s location provided in Figure 13. All devices are illustrated in Figure 17, with a detailed connection diagram in Figure 18. The TA signal collection system uses the through mode, with one transducer recording the measurements and the target acting as the sound source. The relationship between temperature and TA signal was established through the analysis of the TA measurements.

**Figure 19:**
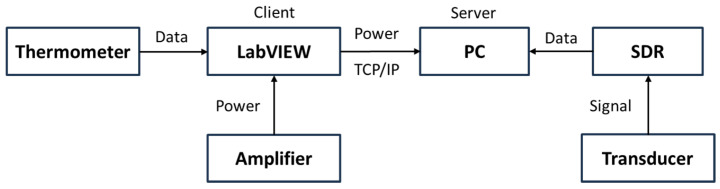
Data collection process.

Experimental data collection was controlled using LabVIEW (v2020) and Python 3.8 scripts integrated with a software-defined radio (SDR). The real-time temperature data collected by sensors was sent to LabVIEW and the TA signal raw data was sent to the SDR. Finally, the temperature and thermoacoustic data were transferred to MATLAB (R2023b) through a TCP/IP communication system for post-processing. A diagram of this data collection process is shown in Figure 19. To verify the reliability of the signal, for each temperature, a constant recording was conducted. For instance, 200,000 times recording results which were extracted from peak–peak amplitude of raw signal shown a stable variance that only had 3% deviation.

**Figure 20:**
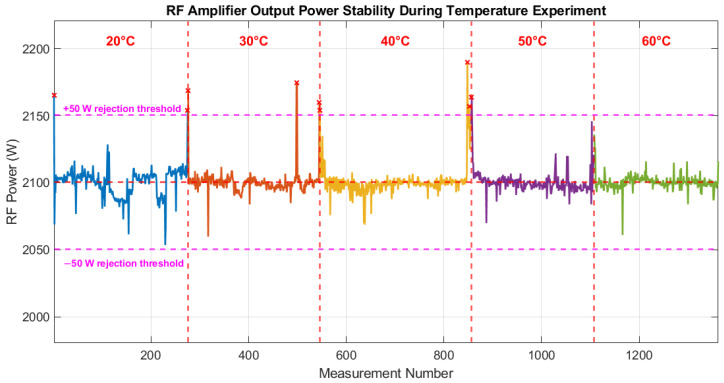
RF power stability during thermoacoustic data acquisition.

To evaluate the stability of the microwave excitation source, RF power measurements were continuously recorded during the experiments. For each temperature condition, at least 200 power measurements were collected and analyzed. Figure 20 shows the recorded power levels during the experiments. To ensure measurement consistency, a power rejection threshold of ±50 W relative to the target operating power was applied. Any thermoacoustic measurements acquired when the recorded power exceeded this threshold were automatically discarded and excluded from further analysis. The results indicate that the microwave excitation power remained stable throughout the data collection process, thereby minimizing uncertainties associated with source amplitude fluctuations.

The acoustic coupling between the transducer and the oil tank was maintained using coupling oil applied between the transducer surface and the acrylic container wall. Since the transducer was rigidly mounted throughout the experiments, no relative movement or geometric changes occurred during data acquisition. To further ensure consistent coupling conditions, a small amount of coupling oil was reapplied approximately every five minutes during the experiments. Consequently, the influence of coupling variations on the measured thermoacoustic signals is expected to be minimal.

The relative positions of the transducer, waveguide, oil tank, and U-shaped target were fixed throughout all experiments. Once the experimental setup was assembled, no repositioning was performed between measurements at different temperatures. Therefore, alignment-related uncertainties were minimized and remained constant for all experiments.

To verify measurement repeatability, a large number of thermoacoustic signals were collected at each temperature condition. The peak-to-peak amplitudes extracted from repeated measurements exhibited less than 3% variation, indicating good signal stability and repeatability. In addition, the experimentally measured microwave power was used to normalize the thermoacoustic amplitudes during post-processing. This normalization compensates for small variations in excitation power and enables a direct comparison between measurements obtained at different temperatures.

## 3. Results

**Figure 21:**
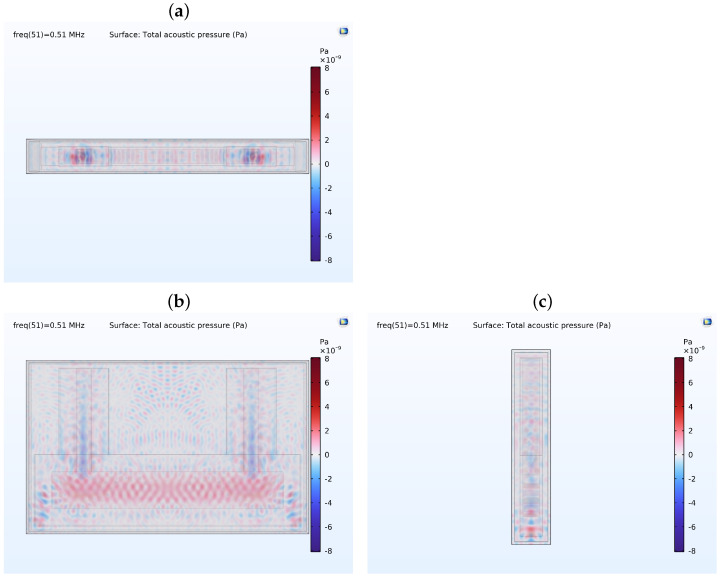
(**a**) Top plane of acoustic pressure at the ROI; (**b**) Front plane of acoustic pressure at the ROI; (**c**) Side plane of acoustic pressure at the ROI.

**Figure 22:**
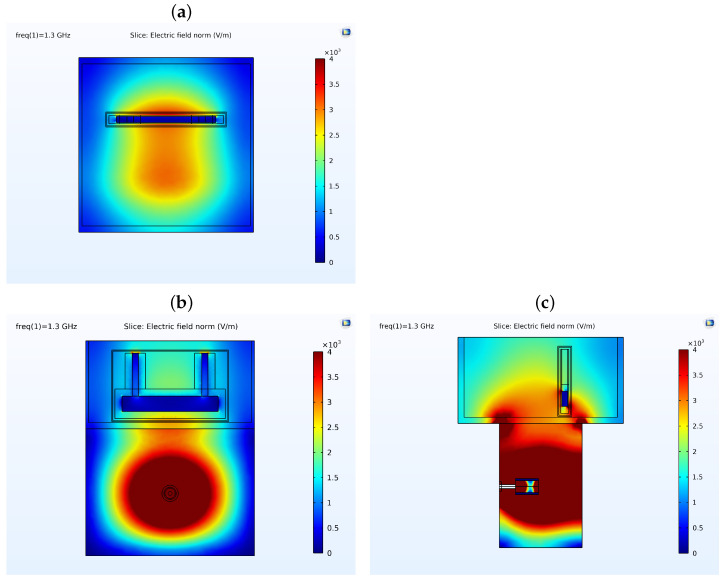
(**a**) Top plane of measurement of electric field; (**b**) Front plane of measurement of electric field; (**c**) Side plane of measurement of electric field.

The solutions to Equations (1) through (5) are illustrated in the following simulation results: the real part of the acoustic pressure in the region of interest across three planes (Figure 21), and the magnitude of the electric field throughout the entire geometry in the same three planes (Figure 22). In the acoustic pressure results, a distinct U-shaped pattern is observed. Additionally, the electromagnetic wave effectively illuminates the sample, and the resulting thermoacoustic (TA) wave emission is clearly visible. These observations indicate that the simulation results are credible and align well with expected physical behavior.

The experimentally acquired thermoacoustic signals were first normalized using the average microwave power measured during each temperature collection stage. This normalization compensates for small variations in microwave excitation power between measurements and enables a direct comparison of thermoacoustic responses obtained at different temperatures. Before power normalization, abnormal RF power records were removed. Specifically, measurements with RF power values deviating by more than ±50W from the mean power value of the corresponding temperature stage were excluded to reduce the influence of unstable microwave excitation. In addition, the experimentally measured attenuation factors presented in Section 2.3 were applied to the simulated frequency-domain responses to account for acoustic propagation losses within the porous medium.

**Figure 23:**
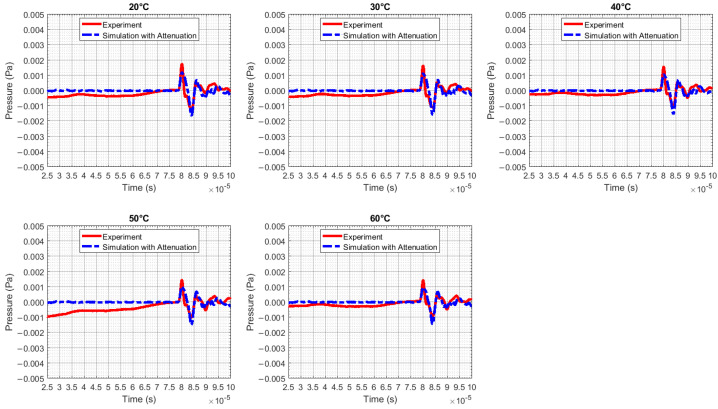
Comparison between simulated and experimentally measured thermoacoustic signals at different temperatures after applying the temperature-dependent dispersion and attenuation corrections.

Figure 23 presents the thermoacoustic signals obtained from both simulations and experiments at different temperatures. Good agreement is observed in both waveform shape and signal arrival time across the investigated temperature range. As the temperature increases, the thermoacoustic signal amplitude decreases in both the experimental measurements and simulation results. This agreement should be interpreted in the context of the calibration procedures used in the model. Specifically, the experimentally calibrated phase velocity was incorporated into the simulation to represent acoustic propagation in the water-saturated sand, and the experimentally measured attenuation correction was applied to the simulated frequency-domain responses. Therefore, the agreement in waveform shape and arrival time demonstrates calibrated agreement between simulation and experiment rather than fully independent predictive validation.

To compare the thermoacoustic amplitudes quantitatively, the peak-to-peak amplitudes of the simulated and experimental signals were extracted. Because the simulations represent an idealized thermoacoustic source while the experiments include additional system sensitivities, a global sensitivity factor was introduced to align the overall amplitude scales. This factor accounts for system-level amplitude effects that are not explicitly modeled, including transducer sensitivity, acoustic coupling efficiency, receiver gain, and data-acquisition response. The sensitivity factor was calculated as the ratio between the mean experimental amplitude and the mean simulated amplitude over all investigated temperatures:

(33) $$S_{f}=\frac{\mathrm{mean}(A_{\exp})}{\mathrm{mean}(A_{\mathrm{sim}})}$$

where $A_{\exp}$ and $A_{\mathrm{sim}}$ denote the peak-to-peak amplitudes extracted from the experimental and simulated thermoacoustic signals, respectively. The simulated amplitudes were subsequently multiplied by this sensitivity factor before comparison.

**Figure 24:**
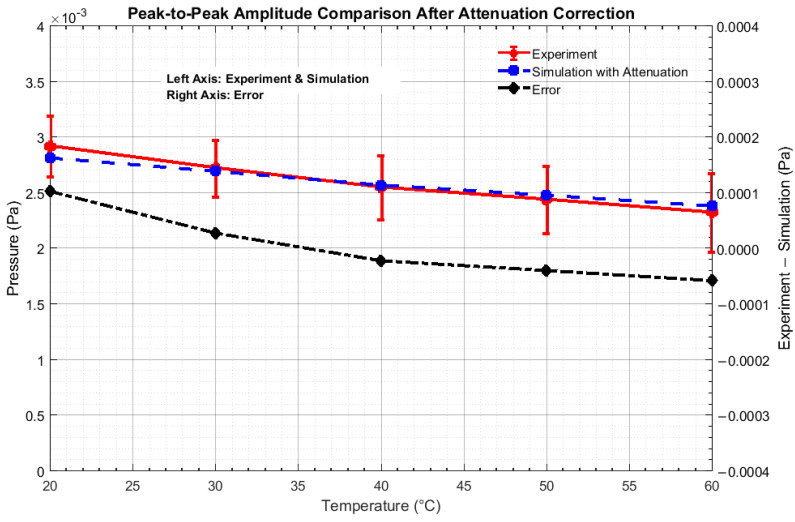
Comparison of thermoacoustic amplitudes obtained from simulations and experiments at different temperatures. Error bars represent the standard deviation of repeated measurements.

Figure 24 compares the peak-to-peak thermoacoustic amplitudes obtained from simulations and experiments over the temperature range from 20 °C to 60 °C. After incorporating the temperature-dependent thermal expansion coefficient, experimentally calibrated phase velocity, attenuation correction, and global sensitivity factor, the simulated amplitudes closely follow the experimental trend. Both datasets show a monotonic decrease in thermoacoustic amplitude with increasing temperature, demonstrating the strong influence of temperature on thermoacoustic signal generation in water-saturated porous media. Since the reported agreement depends on experimentally calibrated phase velocity, attenuation correction, and the global sensitivity factor, it should be regarded as calibrated model validation rather than fully independent predictive validation. The calibrated model reproduces the measured waveform, arrival time, and temperature-dependent amplitude trend under the present laboratory conditions; however, the present comparison should not be interpreted as an independent prediction without experimental calibration.

The decreasing thermoacoustic amplitude can be explained by the combined temperature dependence of thermoelastic expansion, electromagnetic power deposition, and acoustic propagation loss. According to the thermoacoustic source expression in Equation (15), the monopole source term is proportional to $\beta Q_{\mathrm{loss}}/\left(C_{p}\rho \right)$, where β is the effective thermal expansion coefficient, $C_{p}$ is the specific heat capacity, ρ is the density, and $Q_{\mathrm{loss}}$ is the electromagnetic power dissipation density. Although the effective thermal expansion coefficient increases with temperature, the received thermoacoustic amplitude is not governed by β alone. The electromagnetic loss term $Q_{\mathrm{loss}}$ depends on the effective electrical conductivity and the local electric field distribution, which are both affected by the temperature-dependent dielectric properties of the water-saturated sand. As temperature increases, the effective electrical conductivity decreases, while the local electric field distribution and acoustic attenuation also vary with temperature. Therefore, the deposited electromagnetic energy and received acoustic pressure can decrease even though the effective thermal expansion coefficient increases.

The remaining differences between simulations and experiments are attributed primarily to uncertainties in porous medium properties, acoustic coupling conditions, microwave power fluctuations, and temperature measurement accuracy. Nevertheless, the overall calibrated agreement confirms that the proposed modeling framework successfully captures the dominant temperature-dependent thermoacoustic mechanisms and can be used to estimate subsurface temperature variations in water-saturated porous media under controlled laboratory conditions.

## 4. Discussion

A key contribution of this work is the development of a non-contact methodology for monitoring temperature-dependent thermoacoustic responses in porous media. By incorporating rock physics models, experimentally measured thermal expansion coefficients, and temperature-dependent acoustic properties into a multiphysics simulation framework, a quantitative relationship between thermoacoustic signal amplitude and temperature can be established for a given porous medium. It should be emphasized that this relationship is established through a calibrated modeling framework rather than through a fully independent predictive model. Once calibrated, this relationship enables remote estimation of bulk temperature variations based on measured thermoacoustic signals without the need for invasive probes or direct physical access. Compared with conventional techniques such as electrical resistivity tomography, distributed temperature sensing, and infrared imaging, the proposed approach combines electromagnetic excitation and acoustic detection to provide temperature-sensitive measurements within porous media while maintaining a non-contact sensing configuration.

The present validation should therefore be interpreted within the context of the calibration procedures used in this study. The experimentally measured phase velocity was incorporated into the numerical model to represent the frequency-dependent acoustic propagation behavior of the porous medium, while the experimentally measured attenuation factor was applied to the simulated frequency-domain response during post-processing. In addition, a global sensitivity factor was used to account for system-level amplitude effects that were not explicitly modeled, including transducer sensitivity, acoustic coupling efficiency, receiver gain, and data-acquisition response. As a result, the comparison between simulations and experiments demonstrates calibrated agreement under controlled laboratory conditions. This calibrated agreement is useful for verifying the dominant EM–thermal–acoustic mechanisms and the measured temperature-dependent amplitude trend, but it does not yet constitute fully independent predictive validation. Future work should reduce the dependence on empirical amplitude calibration by independently characterizing the system transfer function, acoustic coupling efficiency, and temperature-dependent material properties.

The quantitative uncertainty in the measured thermoacoustic amplitude was evaluated statistically from repeated measurements. For each temperature condition, more than 200 repeated thermoacoustic acquisitions were collected, and each acquisition was obtained by averaging 1000 triggered raw waveforms. Therefore, more than $2.0\times 10^{5}$ triggered waveforms contributed to the measured thermoacoustic response at each temperature, which substantially reduced random waveform noise. For the amplitude comparison, the peak-to-peak amplitude was extracted from each repeated acquisition, and the mean value was used as the representative thermoacoustic amplitude at that temperature. The variation of these repeated peak-to-peak amplitudes was used as the error bar in Figure 24, representing the repeatability of the measurement. In addition, RF power instability was reduced by removing abnormal microwave power records before normalization. Specifically, RF power values outside the mean power value of each temperature stage by more than ±50W were excluded, and the remaining thermoacoustic amplitudes were normalized using the average microwave power of the corresponding temperature stage. Temperature-control uncertainty was reduced by allowing each temperature stage to stabilize for approximately 30–40 min before data acquisition. The porosity of the water-saturated sand was measured from repeated volumetric saturation tests to be approximately 30%, with values mainly ranging from 29% to 31%, corresponding to an estimated uncertainty of about ±1%. This porosity uncertainty propagates into the calculated effective dielectric, thermal, and elastic properties of the porous medium. Additional systematic uncertainties may arise from acoustic coupling, transducer alignment, attenuation calibration, and the global sensitivity factor used to match the simulation and experimental amplitude scales. Therefore, the reported simulation–experiment agreement should be interpreted as calibrated agreement within the measured experimental uncertainty rather than as uncertainty-free independent predictive validation.

The broader implications of this work are particularly relevant to in situ chemical oxidation (ISCO) applications. ISCO remediation processes are typically accompanied by exothermic chemical reactions that generate measurable temperature changes within the subsurface. Consequently, temperature can serve as an indirect indicator of reaction activity and remediation effectiveness. The results presented in this study suggest that thermoacoustic measurements may provide a complementary approach for monitoring these temperature variations and evaluating treatment progress.

However, several challenges must be addressed before field-scale implementation becomes feasible. First, electromagnetic penetration depth may become a limiting factor in practical subsurface environments. The experiments presented in this work were conducted at laboratory scale using relatively short propagation distances and a controlled porous medium. In field applications, electromagnetic attenuation may increase significantly due to larger propagation distances, higher water saturation, elevated electrical conductivity, and the presence of clay-rich formations. Future investigations should therefore evaluate the trade-off between penetration depth and thermoacoustic generation efficiency at different excitation frequencies.

Second, natural geological formations are considerably more heterogeneous than the homogeneous water-saturated sand investigated in this study. Variations in porosity, mineral composition, permeability, fluid saturation, and contaminant concentration can influence both thermoacoustic generation and acoustic propagation. In addition, porous media containing multiple fluid phases, such as water–oil mixtures, may exhibit significantly different thermoacoustic responses because the effective dielectric, thermal, and acoustic properties depend on the fluid composition. Since water generally exhibits substantially higher microwave absorption than most hydrocarbon fluids, variations in fluid composition can alter the deposited electromagnetic energy and consequently affect the generated thermoacoustic signal amplitude. Nevertheless, the proposed modeling framework is fundamentally based on material-property-driven multiphysics simulations and can therefore be extended to such systems by incorporating appropriate effective-medium models and fluid-dependent material properties.

Similarly, non-uniform water saturation may introduce spatial variations in dielectric permittivity, thermal expansion coefficient, density, and acoustic velocity, resulting in heterogeneous thermoacoustic generation and propagation characteristics. These variations may influence both the amplitude and waveform of the measured thermoacoustic signals. However, because the proposed methodology directly links thermoacoustic signal generation to the local material properties, it remains applicable to partially saturated porous media provided that spatially varying saturation distributions and the corresponding effective properties are incorporated into the numerical model. Therefore, although the current study is limited to homogeneous water-saturated sand, the proposed framework is not restricted to this specific medium and can be extended to more complex geological environments in future investigations.

It is also important to clarify the scope of the present work. The experiments were conducted under approximately uniform temperature conditions, and the measured thermoacoustic responses therefore represent bulk temperature-dependent behavior of the porous medium. While the results demonstrate that thermoacoustic signals can be used to estimate temperature variations, the current study does not reconstruct spatially varying temperature distributions. Instead, the work establishes and validates the underlying relationship between temperature and thermoacoustic signal generation in porous media. Achieving true temperature-field reconstruction would require multiple sensing locations, tomographic reconstruction techniques, or array-based acquisition systems capable of resolving temperature-dependent thermoacoustic responses throughout a volume. Such developments represent an important direction for future research.

Compared with existing ISCO monitoring techniques, the proposed thermoacoustic approach offers several potential advantages and limitations. Thermocouples and conventional temperature sensors provide accurate measurements but only at discrete locations. Distributed temperature sensing systems can provide continuous temperature profiles but typically require permanent borehole installations and specialized optical instrumentation. Electrical resistivity tomography offers larger spatial coverage but infers temperature indirectly through conductivity changes. In contrast, thermoacoustic sensing directly exploits temperature-dependent electromagnetic and thermoelastic properties and may therefore provide an additional source of information regarding subsurface thermal behavior. Nevertheless, further studies are required to evaluate its performance under realistic field conditions, particularly in heterogeneous formations with complex fluid compositions and variable saturation conditions.

Despite these limitations, the calibrated agreement between the experimental measurements and the multiphysics simulations demonstrates that the dominant temperature-dependent mechanisms governing thermoacoustic signal generation are captured by the proposed framework. The incorporation of experimentally measured thermal expansion coefficients, acoustic dispersion, and attenuation properties significantly improves the physical consistency of the model and establishes a foundation for future thermoacoustic monitoring applications in porous media. However, future studies should further validate the predictive capability of the framework using independently measured system parameters and more complex porous media conditions.

## 5. Conclusions

This study demonstrates that thermoacoustic (TA) pressure waves can be effectively utilized as a non-contact sensing mechanism for monitoring temperature-dependent behavior in water-saturated porous media. A clear relationship between thermoacoustic signal amplitude and temperature was established through a combination of controlled laboratory experiments and multiphysics simulations. The agreement between experimental measurements and simulation results should be interpreted as calibrated agreement rather than fully independent predictive validation, because the model incorporated experimentally calibrated phase velocity, attenuation correction, and a global sensitivity factor. Within this calibrated framework, the proposed model successfully captures the dominant temperature-dependent mechanisms governing thermoacoustic wave generation and propagation.

The numerical model incorporates temperature-dependent material properties derived from rock physics formulations, experimentally measured thermal expansion coefficients based on Kell’s thermophysical data, and acoustic dispersion and attenuation calibrations obtained from experimental measurements. In addition, a global sensitivity factor was used to align the amplitude scale between the idealized numerical model and the experimental measurement system. By integrating these temperature-dependent and experimentally calibrated parameters into the simulation framework, good agreement was achieved between the simulated and measured thermoacoustic responses over the investigated temperature range from 20 °C to 60 °C. Therefore, the present results validate the calibrated multiphysics framework under controlled laboratory conditions, but they should not be interpreted as a fully independent prediction without experimental calibration.

The results demonstrate that thermoacoustic signals can be used to estimate temperature variations in water-saturated porous media without direct physical contact. The proposed methodology therefore provides a foundation for non-invasive temperature monitoring in applications involving heat transport and temperature-dependent processes within porous materials. However, for practical predictive use, the dependence on experimentally calibrated acoustic parameters and amplitude scaling should be further reduced by independently characterizing the system transfer function, transducer response, acoustic coupling efficiency, and temperature-dependent porous-medium properties.

The potential relevance of this work extends to in situ chemical oxidation (ISCO), geothermal systems, groundwater studies, and other subsurface processes where temperature serves as an important indicator of physical or chemical activity. Although the present study was conducted under controlled laboratory conditions using a relatively homogeneous porous medium, the results establish the feasibility of thermoacoustic temperature monitoring and provide a framework for future investigations under more realistic field conditions.

Future work will focus on extending the methodology to heterogeneous porous media, evaluating performance under field-relevant conditions, and investigating multi-sensor configurations capable of providing spatially resolved thermoacoustic measurements. Additional studies on electromagnetic penetration, acoustic propagation in complex geological materials, large-scale deployment, and independent predictive validation using separately measured system and material parameters will further support the development of thermoacoustic sensing technologies for subsurface environmental monitoring.

In conclusion, thermoacoustic sensing represents a promising approach for non-invasive temperature monitoring in porous media. By combining experimental validation, rock physics modeling, and temperature-dependent acoustic characterization, this work establishes a physically consistent framework for thermoacoustic temperature sensing and provides a foundation for future advances in subsurface environmental monitoring technologies. The current study demonstrates calibrated model validation and temperature-trend estimation under controlled laboratory conditions, while fully independent predictive validation remains an important objective for future work.

## Figures and Tables

**Table 1:** Sand properties assumed temperature-independent over 20–60 °C.

Sand Temp. (°C)	ϵ (-)	ρ ($\mathrm{kg}/m^{3}$)	*k* (W/(m·K))	σ (S/m)	$C_{p}$ (J/(kg·K))	β (1/K)	$C_{l}$ (m/s)	$C_{s}$ (m/s)
20–60	4.06	2650	0.1768	0.0257	743	$9.5\times 10^{-9}$	2008	1075

**Table 2:** Water properties used in simulations.

Water Temp. (°C)	ϵ (-)	ρ ($\mathrm{kg}/m^{3}$)	*k* (W/(m·K))	σ (S/m)	$C_{p}$ (J/(kg·K))	β (1/K)	$C_{l}$ (m/s)	$C_{s}$ (m/s)
20	79	998.2	0.59	0.3976	4183	$2.07\times 10^{-4}$	1481	0
30	76	995.7	0.61	0.3365	4174	$3.03\times 10^{-4}$	1507	0
40	73	992.2	0.63	0.2675	4174	$3.85\times 10^{-4}$	1526	0
50	70	988.1	0.65	0.2455	4174	$4.58\times 10^{-4}$	1541	0
60	67	983.2	0.655	0.2235	4179	$5.23\times 10^{-4}$	1552	0

**Table 3:** Repeated porosity measurements using the volumetric saturation method.

Trial	Injected Water Volume (mL)	Porosity (%)
1	3.0	30
2	3.1	31
3	3.0	30
4	2.9	29
5	3.0	30
6	3.1	31
7	3.0	30
8	2.9	29
9	3.0	30
10	3.0	30
Mean	3.0	30.0

**Table 4:** Elastic properties of water-saturated sand with 30% porosity.

Temperature (°C)	Water $K_{w}$ (GPa)	Sand $K_{s}$ (GPa)	Sand $\mu_{s}$ (GPa)	Effective Porous Medium $K_{\mathrm{eff}}$ (GPa)	Effective Porous Medium $\mu_{\mathrm{eff}}$ (GPa)
20	2.189	6.602	3.062	4.349	0.664
30	2.261	6.602	3.062	4.411	0.664
40	2.311	6.602	3.062	4.453	0.664
50	2.346	6.602	3.062	4.482	0.664
60	2.368	6.602	3.062	4.500	0.664

**Table 5:** Effective target properties of water-saturated sand computed using the rock physics model with 30% porosity.

Target Temp. (°C)	ϵ (-)	ρ ($\mathrm{kg}/m^{3}$)	*k* (W/(m·K))	σ (S/m)	$C_{p}$ (J/(kg·K))	β (1/K)	$C_{l}$ (m/s)	$C_{s}$ (m/s)
20	17.720	2154.5	0.590	0.09498	1221.1	$2.58\times 10^{-5}$	1558.7	555
30	17.216	2153.7	0.610	0.07845	1218.9	$3.88\times 10^{-5}$	1568.2	555
40	16.712	2152.7	0.630	0.06527	1217.4	$5.02\times 10^{-5}$	1574.7	555
50	16.207	2151.4	0.650	0.04534	1215.7	$6.06\times 10^{-5}$	1579.5	555
60	15.701	2150.0	0.655	0.03819	1214.4	$6.97\times 10^{-5}$	1582.7	555

## Data Availability

The experiment data and processing code are available on the Zenodo platform with free access. DOI: 10.5281/zenodo.18522141.
